# A Novel Microbial Dysbiosis Index and Intestinal Microbiota-Associated Markers as Tools of Precision Medicine in Inflammatory Bowel Disease Paediatric Patients

**DOI:** 10.3390/ijms25179618

**Published:** 2024-09-05

**Authors:** Francesca Toto, Chiara Marangelo, Matteo Scanu, Paola De Angelis, Sara Isoldi, Maria Teresa Abreu, Salvatore Cucchiara, Laura Stronati, Federica Del Chierico, Lorenza Putignani

**Affiliations:** 1 Immunology, Rheumatology and Infectious Diseases Research Area, Unit of Microbiome, Bambino Gesù Children’s Hospital, IRCCS, 00165 Rome, Italy; francesca.toto@opbg.net (F.T.); chiara.marangelo@opbg.net (C.M.); matteo.scanu@opbg.net (M.S.); 2 Digestive Endoscopy and Surgery Unit, Bambino Gesù Children’s Hospital, IRCCS, 00165 Rome, Italy; paola.deangelis@opbg.net; 3 Pediatric Gastroenterology and Hepatology Unit, Santobono-Pausilipon Children’s Hospital, 80122 Naples, Italy; isoldi.sara@gmail.com; 4 Crohn’s and Colitis Center, Division of Digestive Health and Liver Diseases, Department of Medicine, University of Miami Miller School of Medicine, Miami, FL 33136, USA; mabreu1@med.miami.edu; 5 Maternal Child Health Department, Pediatric Gastroenterology and Liver Unit, Sapienza University of Rome, 00185 Rome, Italy; salvatore.cucchiara@uniroma1.it; 6 Department of Molecular Medicine, Sapienza University of Rome, 00185 Rome, Italy; laura.stronati@uniroma1.it; 7 Unit of Microbiology and Diagnostic Immunology, Unit of Microbiomics and Immunology, Rheumatology and Infectious Diseases Research Area, Unit of Microbiome, Bambino Gesù Children’s Hospital, IRCCS, 00165 Rome, Italy; lorenza.putignani@opbg.net

**Keywords:** inflammatory bowel disease (IBD), microbial dysbiosis index (MDI), biomarkers, metabolic dysbiosis, gut microbiota, intestinal permeability, mucosal immunity activation, disease severity

## Abstract

Recent evidence indicates that the gut microbiota (GM) has a significant impact on the inflammatory bowel disease (IBD) progression. Our aim was to investigate the GM profiles, the Microbial Dysbiosis Index (MDI) and the intestinal microbiota-associated markers in relation to IBD clinical characteristics and disease state. We performed 16S rRNA metataxonomy on both stools and ileal biopsies, metabolic dysbiosis tests on urine and intestinal permeability and mucosal immunity activation tests on the stools of 35 IBD paediatric patients. On the GM profile, we assigned the MDI to each patient. In the statistical analyses, the MDI was correlated with clinical parameters and intestinal microbial-associated markers. In IBD patients with high MDI, Gemellaceae and Enterobacteriaceae were increased in stools, and *Fusobacterium*, *Haemophilus* and *Veillonella* were increased in ileal biopsies. Ruminococcaceae and WAL_1855D were enriched in active disease condition; the last one was also positively correlated to MDI. Furthermore, the MDI results correlated with PUCAI and Matts scores in ulcerative colitis patients (UC). Finally, in our patients, we detected metabolic dysbiosis, intestinal permeability and mucosal immunity activation. In conclusion, the MDI showed a strong association with both severity and activity of IBD and a positive correlation with clinical scores, especially in UC. Thus, this evidence could be a useful tool for the diagnosis and prognosis of IBD.

## 1. Introduction

The term IBD (inflammatory bowel disease) refers to a group of chronic immune-mediated inflammatory diseases of the intestinal mucosa [1,2], associated with gut dysbiosis [1,3], including ulcerative colitis (UC) and Crohn’s disease (CD) [1,4,5,6].

In particular, UC affects the rectum and colon and is characterised by distal to proximal and continuous inflammation [5,7]. Lesions are usually diffuse and superficial [5]. Deep ulceration is seen only in patients with severe disease [5]. In the course of the UC, the proximal extent of inflammation progresses to cumulative pancolitis [5]. Ileitis usually remains superficial and does not involve deep ulceration [5]. In addition, colonic lesions may regress and localise to the distal colon [5]. In contrast, the inflammatory process in CD can affect any part of the digestive tract but mainly the distal ileum and colon [5]. The Montreal classification of CD distinguishes between disease at the level of the ileum, the colon, and either the ileum or colon [5]. Moreover, colonoscopy is only a gold standard for diagnosing and screening diseases in the colon and rectum [8]; its widespread use is often hampered by unpleasant experiences and logistical obstacles [9], and patients often suffer from colonoscopy anxiety [10].

Although the aetiology of IBD remains unknown, host genetics, gut microbiota (GM) and the immune system have been implicated [11,12,13,14,15,16]. The IBD risk has been linked to over 240 host genetic loci, most of which are associated with key immunological pathways, including innate immunity, immune responses and autophagy [17,18,19,20]. 

Furthermore, the imbalance in the GM composition in IBD patients has been demonstrated in adults [21,22,23,24,25,26,27] and children [28]. At a deeper level, the gut microbiota fingerprint of paediatric IBD patients is characterised by a decrease in *Eubacterium*, *Lactobacillus*, *Parabacteroides* and *Ruminococcus*, which characterise the gut microbiota of healthy children. On the other hand, it is characterised by an increase in *Actinobacillus*, *Haemophilus*, *Prevotella*, *Streptococcus*, *Veillonella*, *Fusobacterium* and *Enterobacter* and *Escherichia*; the latter go beyond the mucosa to invade the intestinal epithelial cells and trigger the immune response [29]. In particular, *Enterobacter* and *Escherichia* represent biomarkers of IBD in children, but also in adults, suggesting a possible transition from childhood to adulthood [29]. Then, the presence of specific gut microbiota in paediatric subjects without IBD clinical symptoms could be indicative of a dysbiotic gut microbiota that predisposes to the onset of IBD in adulthood [16].

More specifically, gut dysbiosis is defined as a structural and functional alteration of the GM that leads to a disruption of mucosal homeostasis and induces an excessive and continuous activation of immune responses to specific food components and GM factors [4,30,31,32]. Defining dysbiosis is quite difficult; in fact, the GM perturbation can range from a change in a few species to the replacement of entire microbial communities [33].

However, to quantify dysbiosis, several indexes have been proposed to help characterise diseases status and to predict treatment response [34]. To date, the potential causal relationships between intestinal dysbiosis and diseases are not fully captured by any of the dysbiosis indexes [34].

Moreover, defining a gut dysbiosis profile associated with disease activity, localisation and severity in children with IBD could be important in clinical practice for developing more personalised therapies. 

For these reasons, our aim is to present a novel method able to estimate gut dysbiosis associated with IBD. Moreover, by correlating the IBD clinical scores and disease activity with the gut Microbial Dysbiosis Index (MDI), we suggest a potential analytical tool for predicting disease activity and progression. 

## 2. Results

### 2.1. Study Population

A cohort of 35 IBD patients (14 UC and 21 CD patients) was included in this study. The clinical characteristics of all patients in terms of degree of dysbiosis, disease activity, severity and location, treatments and endoscopic scores (i.e., PUCAI, PCDAI, Matts score and SES-CD) are reported in Table 1. 

### 2.2. IBD Faecal Microbiota Compared to Healthy Controls

Comparing the faecal microbiota between IBD and CTRLs, we found a significant increase in the α-diversity, assessed using the Shannon–Weiner index, in the IBD cohort respect to CTRLs (Figure S1A), while the β-diversity, performed using Bray–Curtis dissimilarity, resulted no statistically significant (Figure S1B). The Mann–Whitney U test showed a statistically significant increase in *Haemophilus*, *Streptococcus*, *Eggerthella*, *Ruminococcus*, *Enterococcus*, *Anaerostipes*, *Lactobacillus*, *Sutterella* and *Fusobacterium* and Enterobacteriaceae in IBD patients. On the other hand, *Coprococcus*, *Oscillospira*, *Clostridiales*, *Ruminococcaceae*, *Christensenellaceae*, *Ruminococcaceae Ruminococcus*, *Alistipes*, *Gemmiger*, *Gemellaceae*, *Mogibacteriaceae*, *Barnesiellaceae*, *Parabacteroides Prevotella* and *Akkermansia* were increased in CTRLs (Table S1). 

### 2.3. Gut Dysbiosis in IBD

We tested age, gender and treatments as confounding factors in the faecal microbiota analysis, as shown in Table S2. This analysis excluded the confounding effects of these variables in the GM analysis (Table S2). 

Then, to test how the different levels of intestinal dysbiosis could affect the GM profiles, we stratified the patients according to the percentage of gut MDI, assigning them to the following groups: MDI < 25%, mild dysbiosis; MDI between 25% and 35%, moderate dysbiosis; and MDI > 35%, high dysbiosis.

We obtained 8 (22.9%) patients with a mild MDI, 19 (54.2%) patients with a moderate MDI, 8 (22.9%) patients with a high MDI.

When comparing the faecal microbial ecology of patients stratified according to MDI, we obtained a slight, but not statistically significant, decrease in the Chao1 index in the high-MDI group compared to the low and medium ones (Figure S2A). The β-diversity analysis, assessed using Bray–Curtis dissimilarity, identified three different clusters according to the degree of MDI (PERMANOVA = 0.008) (Figure S2B).

**Figure 1:**
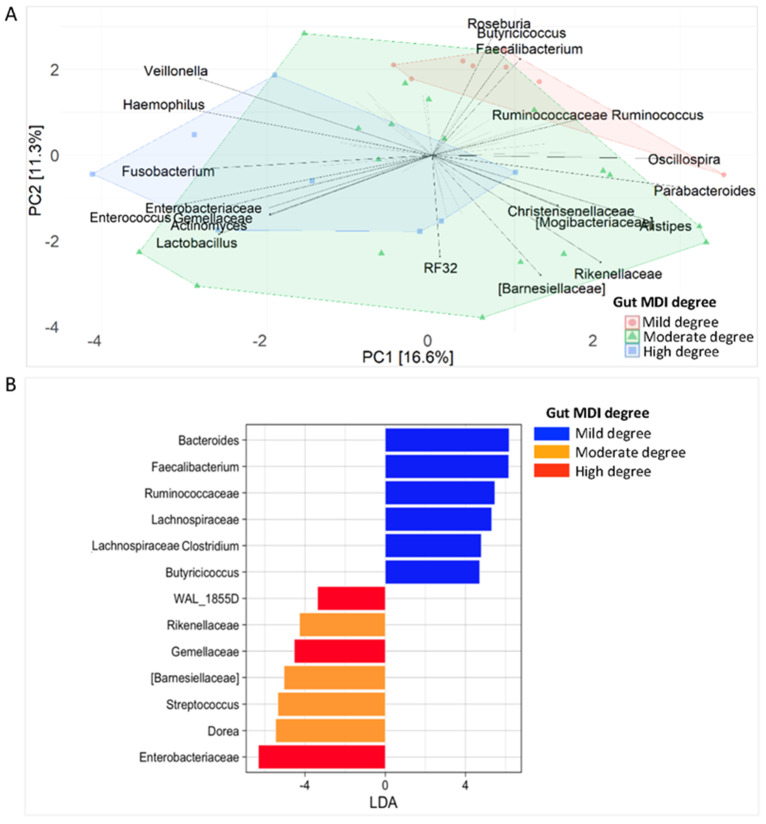
Faecal microbiota fingerprint of 35 IBD patients stratified based on MDI (mild = 8, moderate = 19, high = 8). (**A**) Principal component analysis (PCA) plot for multivariate unsupervised analysis. (**B**) Linear discriminant analysis (LDA) plot on linear discriminant analysis effect size (LEfSe) univariate analysis.

A PCA analysis assigned *Oscillospira*, Ruminococcaceae_*Ruminococcus*, *Faecalibacterium*, *Butyricicoccus* and *Roseburia* to mild MDI, whereas it assigned *Enterococcus*, *Fusobacterium*, *Haemophilus* and *Veillonella* to high MDI (Figure 1A). The moderate-MDI group was characterised by *Parabacteroides*, *Alistipes*, Rikenellaceae, Barnesiellaceae, Christensenellaceae, Mogibacteriaceae, o_RF32 and *Lactobacillus*.

*Bacteroides*, *Faecalibacterium*, Ruminococcacceae, Lachnospiraceae, Lachnospiraceae_*Clostridium* and *Butyricicoccus* were identified as biomarkers of mild MDI. 

WAL_1855D, Gemellaceae and Enterobacteriaceae were assessed as biomarkers of high MDI and Rikenellaceae, Barnesiellaceae, *Streptococcus* and *Dorea* as biomarkers of moderate MDI. Furthermore, integrating multivariate and univariate approaches, Gemellaceae and Enterobacteriaceae were assigned to high MDI, Ruminococcaceae, *Faecalibacterium* and *Butyricicoccus* to mild MDI and Rikenellaceae and Barnesiellaceae to moderate MDI (Figure 1A,B).

### 2.4. Correlation of Gut MDI and GM Profile with Disease Site and State

**Figure 2:**
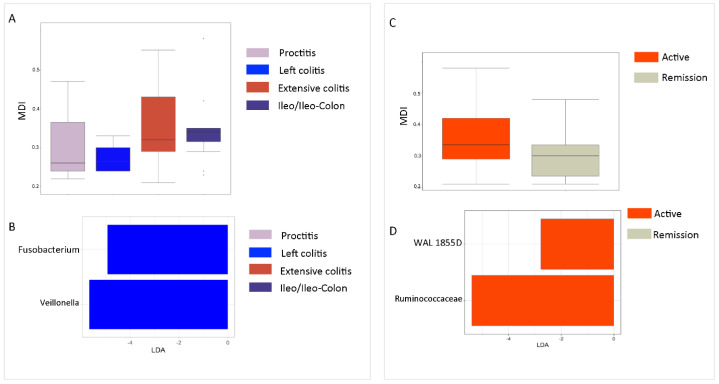
Gut MDI as a function of disease characteristics. (**A**) Histogram of MDI in patients grouped for disease localisation. Kruskal–Wallis *p*-value > 0.05. (**B**) LDA plot on LEfSe univariate analysis applied to GM profiles of patients stratified for disease localisation. (**C**) Histogram of MDI in patients grouped for disease activity status. Mann–Whitney test *p*-value > 0.05. (**D**) LDA plot on LEfSe univariate analysis applied to GM profiles of patients stratified for disease activity status.

By correlating intestinal MDI and disease localisation, an increase in intestinal MDI in IBD patients with extensive colitis and ileo/ileocolon compared to others with proctitis and left colitis was registered (Table S3). A linear discriminant analysis effect size (LEfSe) analysis (Figure 2B) assigned *Fusobacterium* and *Veillonella* as biomarkers of extensive colitis, while for other disease localisations, other bacterial markers were not identified.

Comparing MDI in patients stratified by disease state (i.e., active disease and remission), an increase in MDI was observed for the active disease group (*p* > 0.05), characterised by high distribution of WAL_1855D and Ruminococcaceae (Figure 2C,D).

The regression analysis performed between gut MDI and Chao1 and between gut MDI and faecal calprotectin levels showed the absence of correlations between gut MDI and these two variables (*p*-value = 0.47 and *p* = 0.29, respectively) (Figure S3A,B). 

### 2.5. Gut Dysbiosis in UC and CD

**Figure 3:**
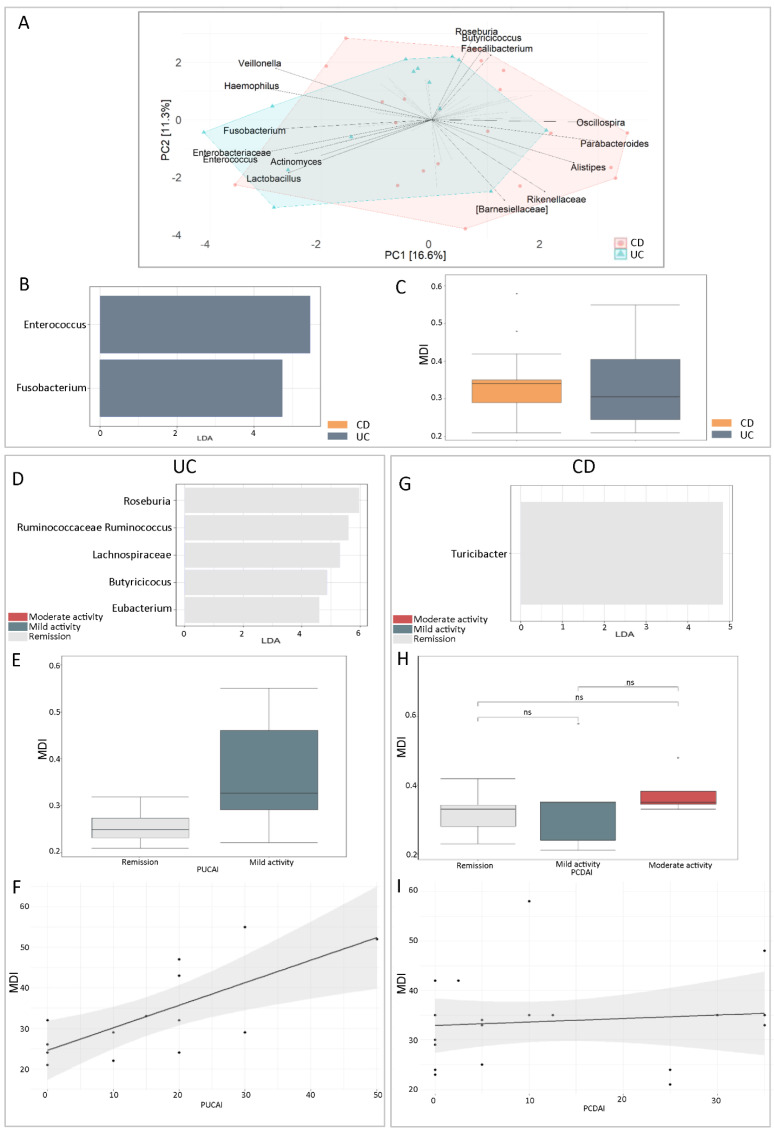
GM profiles associated with UC and CD. (**A**) PCA plot of UC and CD microbiota profiles. (**B**) LDA plot of LEfSe univariate analysis for the comparison between UC and CD microbiota profiles. (**C**) Box plot of intestinal MDI of CD compared with UC. (**D**) LDA plot of LEfSe univariate analysis of microbiota profiles for the comparison of UC patients stratified for disease activity. (**E**) Box plot of the gut MDI of UC patients stratified for disease activity. (Mann–Whitney test *p*-value = 0.1). (**F**) Fitted line plot of intestinal MDI and PUCAI (Paediatric Ulcerative Colitis Activity Index). The regression analysis revealed the presence of correlation between these two variables (R2-value = 0.71; *p*-value = 0.004). Each sample is represented by a dot. (**G**) LDA plot of LEfSe univariate analysis of microbiota profiles for the comparison of CD patients stratified for disease activity. (**H**) Box plot of the gut MDI of CD patients stratified for disease activity (Kruskal–Wallis test *p*-value 0.36). (**I**) Fitted line plot of intestinal MDI and PCDAI (Paediatric Crohn’s Disease Activity Index). The regression analysis revealed the absence of correlation between these two variables (R2-value = 0.11; *p*-value = 0.65). Each sample is represented by a dot.

We investigated the differences in GM profiles between UC and CD patients. A comparison of the GM profiles of these two disease typologies revealed a statistically significant decrease in the Chao1 index (*p*-value = 0.0015) in CD patients compared to UC patients (Figure S4A). However, the PERMANOVA test, applied to the β-diversity distance matrix, performed using Bray–Curtis dissimilarity, did not return statistically significant results, indicating that the samples did not cluster by disease typology (Figure S4B). PCA analysis revealed no differences in the faecal microbiota composition between the two cohorts (Figure 3A). However, LEfSe univariate analysis identified *Enterococcus* and *Fusobacterium* as biomarkers of UC GM (Figure 3B). An increase in MDI was evident in CD compared to UC, although this was not statistically significant (*p* > 0.05) (Figure 3C). 

Based on the PUCAI and PCDAI scores, respectively, for UC and CD, we stratified patients into mild and moderate disease activity and disease remission (Table 1). We assigned microbial biomarkers only to disease remission status for UC and CD. In particular, the GM was enriched in *Roseburia*, Ruminococcaceae_*Ruminococcus*, Lachnospiraceae, *Butyricicoccus* and *Eubacterium* in UC patients (Figure 3D) and in *Turicibacter* (Figure 3G) in CD patients. Comparing the gut MDI of patients stratified by disease activity, we obtained a statistically significant increase in MDI in UC patients with mild disease activity compared to those in remission (Figure 3E), but no statistical difference in CD patients (Figure 3H). Moreover, there was no statistically significant correlation between gut MDI and the disease activity index (Figure 3F,I). 

Finally, we also correlated gut MDI with Matts and SES scores for UC and CD, respectively, obtaining a statistically significant positive correlation between MDI and the Matts score (Figure S5A) but none between MDI and SES score (Figure S5B).

### 2.6. Metabolic Biomarker Associated with MDI in IBD

**Figure 4:**
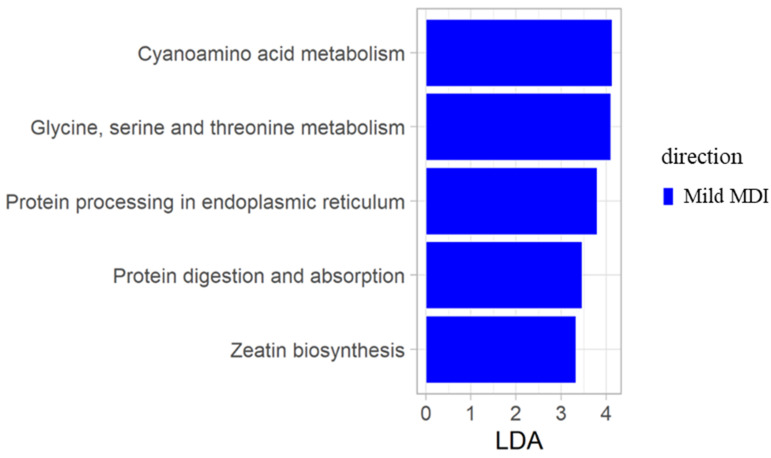
PICRUSt2 functional prediction using the KEGG pathway database. Metabolic biomarkers associated with mild dysbiosis in IBD. LEfSe analysis was performed (LDA score > 3.3).

We performed a functional pathway prediction analysis by applying the PICRUSt2 algorithm to the composition of the faecal microbiota (Figure 4). The results of the KEGG assays indicated that mild dysbiosis was mainly associated with the upregulation of functional pathways belonging to amino acid metabolism, including cyanoamino acid metabolism and the metabolism of glycine, serine and threonine and three other metabolic pathways, including protein processing in the endoplasmic reticulum, protein digestion and absorption and zeatin biosynthesis. No metabolic pathways were associated with a moderate and high degree of dysbiosis. 

### 2.7. Ileal Microbiota Fingerprint in IBD

**Figure 5:**
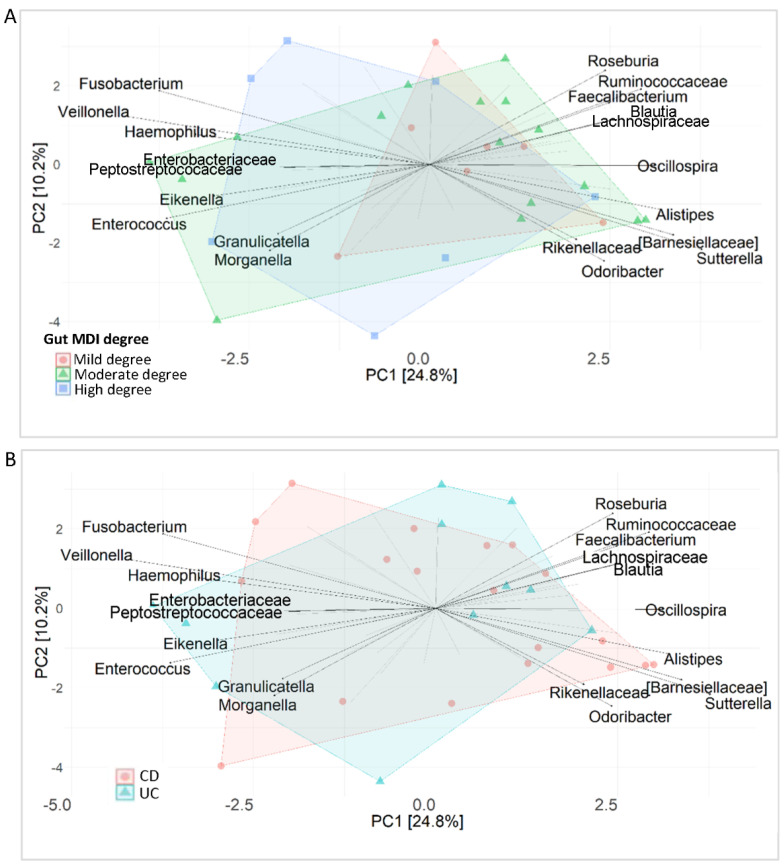
Principal component analysis (PCA) plot for multivariate unsupervised analysis for ileal IBD microbiota profile. (**A**) Ileal microbiota stratified for dysbiosis degree. (**B**) Ileal microbiota in CD and in UC.

The multivariate analysis of metataxonomic data of mucosal microbiota revealed that *Fusobacterium*, *Haemophilus* and *Veillonella* were associated with high dysbiosis, while moderate dysbiosis was characterised by the increase in *Peptostreptococcus*, *Enterobacteriaceae*, *Eikenella*, *Enterococcus*, *Roseburia*, Ruminococcaceae, *Faecalibacterium*, *Lachnospiraceae*, *Oscillospira*, *Alistipes*, Barnesiellaceae, *Sutterella*, *Rikenellaceae* and *Odoribacter* (Figure 5A).

Grouping patients in CD and UC, we showed that *Fusobacterium*, *Haemophilus*, *Veillonella*, *Oscillospira*, *Alistipes*, Barnesiellaceae, *Sutterella*, Rikenellaceae and *Odoribacter* characterised the CD ileal microbiota, whereas *Peptostreptococcus*, Enterobacteriaceae, *Eikenella*, *Enterococcus*, *Roseburia*, Ruminococcaceae, *Faecalibacterium*, and Lachnospiraceae characterised the UC ileal microbiota (Figure 5B). 

The univariate analysis showed an absence of statistically significant differences when comparing patients by the dysbiosis index and by disease typology.

### 2.8. Network between Faecal and Mucosal Microbiota 

**Figure 6:**
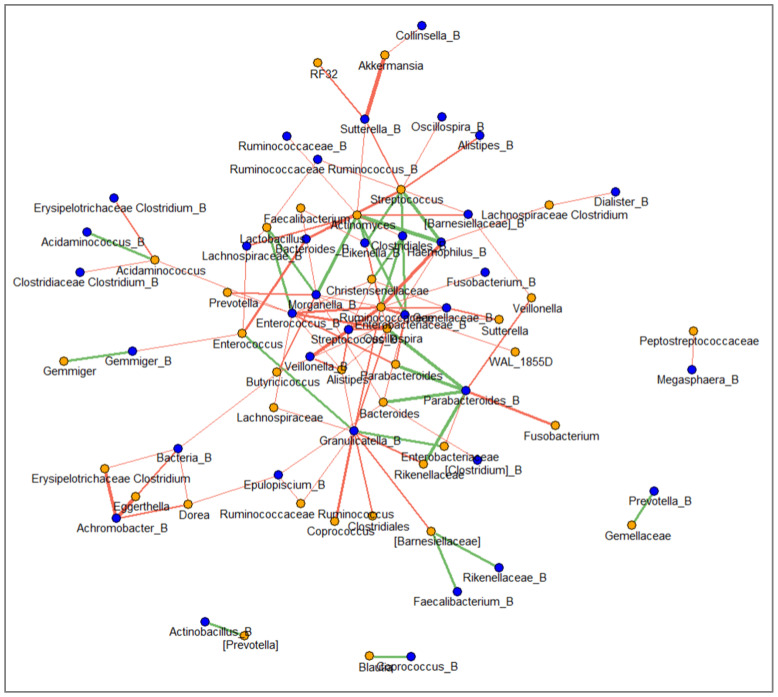
Spearman’s correlation analysis between genus level in faecal microbiota and ileal microbiota. Each node refers to faecal bacteria (orange circles) and ileal bacteria (blue circles). Green and red edges indicate positive and negative correlation values, respectively (filtered to *p*-value < 0.05).

To deepen the relationship between faecal and ileal taxa and to gain a more complete understanding of the gut bacterial ecosystems, we performed a network analysis between faecal and ileal bacteria (Figure 6). The network was characterised by 72 nodes connected by 111 edges. The clustering coefficients ranged from −0.71 to 0.7. The strongest positive correlation was between faecal *Haemophilus* and mucosal *Actinomyces* (rho-value = 0.7). Conversely, the strongest negative correlations were between faecal *Achromobacter* and mucosal *Eggerthella* and between faecal *Sutterella* and mucosal *Akkermansia* (rho-values = −0.71). Selecting the nodes with nine or more edges, we found that amongst ileal bacteria, Enterobacteriaceae, *Enterococcus* and *Granulicatella* established, for the most part, negative connections with faecal bacteria; amongst the ileal bacteria, *Actinomyces*, *Oscillospira*, Ruminococcaceae and *Streptococcus* were interconnected with faecal bacteria through mostly negative connections. 

### 2.9. Correlation between Faecal and Mucosal Bacteria and MDI

Pearson’s correlation test was used to correlate the relative abundances of faecal and ileal microbial taxa with the MDI. The MDI was strongly and positively correlated with faecal Enterobacteriaceae (rho-value = 0.634) and negatively with *Faecalibacterium* (rho-value = −0.537). Interestingly, the MDI results showed—even if moderately—positive correlations with faecal *Fusobacterium*, *Haemophilus* and WAL_1855D and negative correlations with Lachnospiraceae_*Clostridium*, *Bacteroides* and *Butyricicoccus*. Finally, the MDI showed only moderate levels of positive correlations with ileal *Achromobacter*, *Actinobacillus*, *Cloacibacterium*, *Haemophilus*, *Prevotella* and *Pseudomonadaceae* (Table 2).

### 2.10. Metabolic Dysbiosis, Intestinal Permeability and Mucosal Immune Activation in IBD

**Figure 7:**
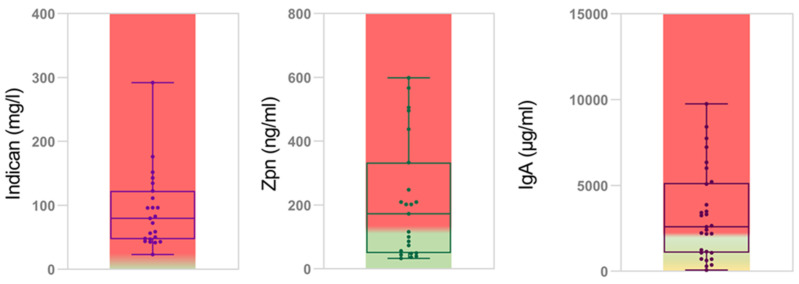
Metabolic dysbiosis, intestinal permeability and mucosal immune activation. Box plots of indican, Zpn and IgA levels measured in the IBD cohort (green for normal levels, yellow for subnormal levels and red for elevated levels). Each sample is represented by a dot.

In the IBD cohort, we analysed the patients’ levels of urinary indican and faecal zonulin, which are markers of metabolic dysbiosis [35] and gut permeability [36,37,38], respectively. The mean indican level ± standard deviation (SD) was 91.77 ± 60.13 mg/L (Figure 7). The physiological range of indican has been described as being from 0 to 10 mg/L [39]. 

The mean zonulin level ± SD was 211.42 ± 186.59 ng/mL (Figure 7). The literature defines for faecal zonulin levels > 107 ng/mL a state of leaky gut and intestinal permeability [40,41]. As for the mucosal immunity parameter, we tested the faecal IgA. The mean IgA level ± SD was 3265.82 ± 2669.03 µg/mL (Figure 7). An IgA range between 510 and 2040 µg/mL is considered physiological [41]. 

We performed the *t*-test on indican, Zpn and IgA levels between UC and CD, but the *p*-values were higher than 0.05. When comparing the levels of indican, Zpn and IgA in IBD patients grouped by mild-, moderate- and high-MDI groups, we did not obtain statistically significant differences (*p*-value > 0.05). (Figure S6). Moreover, the linear regression analysis showed an absence of statistically significant correlations between the MDI values and indican, Zpn and IgA levels (Figure S7A–C). Furthermore, we performed the linear regression analysis between the severity disease scores (PUCAI and PCDAI) and the three markers, but the results of these tests were not statistically significant (Figure S7D–F). The linear regression between the endoscopic scores (Matts score and SES score) did not reveal a statistical significance.

## 3. Discussion

In this study, we have for the first time associated different grades of gut dysbiosis with a specific signature of the GM in IBD. Specifically, we adopted the MDI to stratify patients and to correlate GM modification to disease severity and clinical scores. Moreover, we related metabolic dysbiosis, intestinal permeability and mucosal immunity activation to intestinal dysbiosis.

We identified specific gut microbiota signatures in paediatric patients with IBD when compared to CTRLs. In particular, we assigned an increase in bacterial richness and of *Haemophilus*, *Streptococcus*, *Eggerthella*, *Ruminococcus*, *Enterococcus*, *Anaerostipes*, *Lactobacillus*, *Sutterella*, *Fusobacterium* and Enterobacteriaceae and a decrease in *Coprococcus*, *Oscillospira*, *Clostridiales*, *Ruminococcaceae*, *Christensenellaceae*, *Ruminococcaceae Ruminococcus*, *Alistipes*, *Gemmiger*, *Gemellaceae*, *Mogibacteriaceae*, *Barnesiellaceae*, *Parabacteroides*, *Prevotella* and *Akkermansia* in children with IBD. The increase in *Fusobacterium* and Enterobacteriaceae and the decrease in *Akkermansia* confirmed the inflammatory signature of gut microbiota in IBD. Moreover, in our results, a reduction in microbial richness was observed in IBD patients with high dysbiosis compared to those with mild dysbiosis, consistent with reports in the literature [42,43], suggesting that reduced microbial richness is associated with high levels of IBD inflammation. Furthermore, β-diversity revealed a distinct GM profile in patients with mild dysbiosis and a common profile in those with moderate and high levels of dysbiosis. Specifically, our results showed that Gemellaceae, WAL_1855D and Enterobacteriaceae were increased in the GM of IBD patients with high MDI. In particular, the Gemellaceae family has been found to be a specific biomarker for CD [43,44]. It is also interesting to note that we found that both WAL_1855D and Enterobacteriaceae were positively correlated with MDI. WALD_1855 was also identified in active IBD, suggesting a strong role of both bacterial taxa (WAL_1855D and Enterobacteriaceae) in disease progression. As previously reported in the literature, Enterobacteriaceae are overrepresented in ileoanal pouch biopsies [45] and have been confirmed as a pro-inflammatory biomarker of IBD [46,47,48]. Furthermore, our network revealed that Enterobacteriaceae are negatively correlated with *Parabacteroides*; the latter is known in the literature to play a protective role by improving intestinal epithelial integrity in mouse models of acute and chronic colitis [49,50].

In IBD patients with moderate dysbiosis, Rikenellaceae, Barnesiellaceae, *Streptococcus* and *Dorea* were increased in the GM of this group of patients. In particular, the butyrate-producing family of Rikenellaceae was found to be reduced with UC progression [51,52]. This bacterial family probably has a role in protection of the host against intestinal inflammation and IBD exacerbation. Additionally, Barnesiellaceae and *Streptococcus* are also confirmed to be more abundant in IBD faecal samples, while *Dorea* seems to be decreased in these samples [52,53]. *Dorea* is associated with patients with early CD but decreases in advanced CD [54].

The dysbiotic mucosal bacterial community associated with disease progression was characterised by a relative increase in *Prevotellaceae* and *Pseudomonadaceae* bacteria compared to non-IBD controls [55], consistent with our findings. High levels of *Pseudomonas* and *Achromobacter* have been reported in the literature during the exacerbation phase of UC compared to the remission phase [56]. Furthermore, *Cloacibacterium* was increased in inflamed biopsy in UC patients [57]. *Actinobacillus*, *Pseudomonas* and *Prevotella* were enriched in IBD patients according to our findings. In particular, *Actinobacillus* was associated with CD in intestinal mucosal samples [31].

In IBD patients with mild dysbiosis, we found an increase in Ruminococcaceae, *Faecalibacterium*, *Butyricicoccus*, *Bacteroides*, *Lachnospiraceae* and *Clostridium* (Lachnospiraceae). *Faecalibacterium* and *Butyricicoccus* are producers of SCFAs [58,59], which lead to the suppression of the nuclear factor kb signalling pathway [60], thereby reducing the production of pro-inflammatory cytokines [58,60]. In particular, the immunomodulatory properties of *Faecalibacterium prausnitzii* make it an indicator of gut health and homeostasis [1,61]. Moreover, *Faecalibacterium* and *Butyricicoccus* were negatively correlated with MDI, reinforcing the evidence of their use as potential probiotics for dysbiosis restoration in patients with IBD [62]. It is noteworthy that Lachnospiraceae was increased in UC in remission, showing a positive role against disease progression. In addition, *Bacteroides* and *Clostridium* (Lachnospiraceae) showed an increase in patients with mild dysbiosis and a negative correlation with MDI, confirming the previous evidence of Gevers et al., 2014 [43].

Interestingly, the Ruminococcaceae seemed to have a dubious role. In fact, this microorganism showed an increase in mild dysbiosis patients but also in active diseases. 

*Fusobacterium* and *Haemophilus* are positively correlated with MDI. *Haemophilus*, specifically *H. parainfluenzae*, is an oral commensal bacterium [42,63] but is found to be increased in IBD patients [63,64]. In fact, *Fusobacterium* is closely associated with the development of IBD [65]. In our study, we showed an increase in *Fusobacterium* in UC patients compared to CD patients and we identified *Fusobacterium* as a biomarker for high MDI and extensive colitis, the most severe form of UC. Indeed, the literature confirms that *Fusobacterium* characterises active-phase pancolitis [66] and predisposes one to colorectal cancer (CRC) [67,68,69,70]. Furthermore, as with Enterobacteriaceae, we found that *Fusobacterium* was negatively correlated with *Parabacteroides*, which, in addition to its protective role in the intestinal mucosa (as mentioned above), also has anti-inflammatory effects in colitis, atherosclerosis, type 2 diabetes mellitus, food allergy and obesity [71]. Moreover, *Parabacteroides* has been recognised as the most important probiotic in protecting against CRC and metabolic disorders [70]. Therefore, further knowledge on *Parabacteroides* as a potential future probiotic in IBD therapy is needed. We have also identified *Enterococcus* as a biomarker for UC. *Enterococcus* is abundant in patients with active pouchitis and in patients with active UC [72]. The role of *Enterococcus* in inducing colitis is probably related to its production of bile acids and generation of reactive oxygen species [73].

Regarding the differences between UC and CD, we showed a slight increase in the intestinal MDI in CD compared to UC. Moreover, in UC, this index showed an increase in patients with mild activity compared to those with disease in remission, and it correlated positively with PUCAI and Matts scores, indicating that MDI could be considered for predicting disease activity and severity. Instead, there was no strong association between gut MDI and PCDAI and SES-CD scores in CD patients. These latter findings are in contrast to other studies in which the gut MDI of CD patients was lower than that of UC patients [74] and in which gut MDI and PCDAI were closely correlated [74]. Furthermore, it would be interesting in the future to propose a larger case study and a longitudinal study to confirm the correlation between intestinal MDI and IBD.

Moreover, we also measured the gut metabolic dysbiosis by assaying urinary levels of indican. Indican is a metabolite formed by bacterial cleavage of tryptophan in the gut [75]. Diet, absorption efficiency, the qualitative and quantitative nature of the gut microbiota, the rate of movement of intestinal contents and the frequency of evacuation can influence the amount of urinary indican [75]. In our case series, we observed that the mean urinary indican level in patients was high, indicating a dysbiotic status in IBD. In our analysis, the indican levels and MDI results were not correlated. However, the lack of correlation between these dysbiosis markers could be explained by the dietary origin of urinary indican. 

Furthermore, we also analysed the levels of zonulin, which is a modulator of intercellular tight junction and of intestinal permeability [36,37]. We observed high levels of zonulin, suggesting a leaky gut state in the IBD patients. However, we found no correlation between faecal zonulin levels and MDI, probably due to the low number of available samples. The high mean zonulin levels were due to diet and the fact that there were more CD patients than UC patients. In fact, as the literature suggests, zonulin levels are higher in the faeces of CD patients [40]. 

As the amount of IgA in small faecal samples seems to reflect well the total amount of IgA secreted by the gut [76,77], we also measured the amount of faecal sIgA, which reduces the expression of pro-inflammatory cytokines in the gut. Moreover, sIgA mediates anti-inflammatory functions through interaction with mucosal dendritic cells (DCs). Then, sIgA-antigen complexes taken up by DCs reduce local T-cell activation [78]. Analysis of our results shows a trend in the reduction in IgA in the mild-MDI group compared to the moderate- and high-MDI groups. Moreover, we observed that the majority of the study population has IgA concentrations above the range that would be expected from a relapse of IBD. However, few patients have IgA levels that suggest a deficiency in IgA production.

Regarding the metabolic pathways of faecal microbiota, we found in the mild dysbiosis group an increase in protein processing in the endoplasmic reticulum (ER) pathway. Interestingly, the ER is involved in maintaining the integrity of the intestinal barrier. The lack of intestinal barrier integrity leads to the invasion of pathogens into the intestinal lumen and triggers a series of inflammatory immune responses, characteristic in IBD [79]. Moreover, also the pathway of glycine, serine and threonine metabolism was increased in mild dysbiotic patients. The glycine is involved in the enhancement of the intestinal epithelial barrier by promoting the expression of tight junction proteins by endoplasmic reticulum stress (ERS)-related signalling and by inhibiting ERS-induced apoptosis [79,80]. Indeed, glycine deficiency is associated with oxidative damage and intestinal barrier dysfunction, suggesting a functional role for serine or glycine in intestinal homeostasis [80]. Furthermore, glycine is partly degraded in the liver and partly in the small intestine [79,80]. In fact, glycine is highly incorporated into the proteins of both Gram-positive and Gram-negative intestinal bacteria [80,81,82]. This suggests that glycine is an important amino acid for supporting optimal growth of the GM. Furthermore, our results showed that amino acid metabolism, particularly the cyanoamino acid pathway, was increased in IBD patients with mild dysbiosis. Notably, in gut of patients with systemic lupus erythematosus (SLE), a positive correlation was reported between the cyanoamino acid pathway and *Prevotella* [83,84,85]. This finding was confirmed by our results. In fact, we observed, in mild degree dysbiotic patients, the enrichment of cyanoamino acid and of *Prevotella*. 

Finally, the pathway of protein digestion and absorption was also increased by a mild degree in the dysbiosis group. The malabsorption pathway is involved in proteolytic activity. In the literature, faecal proteolytic activity levels were shown to be elevated in CD patients compared to healthy subjects. The increase in faecal protease levels could be due to the ileal malabsorption and/or to the overgrowth of anaerobic faecal microbiota in CD patients [86]. In addition, faecal bacteria proteases (glycosidases) break down the polymeric structure of mucins [86], causing damage to the intestinal mucosa in CD and UC patients [87]. Finally, high levels of proteolytic enzymes in pouchitis were associated with *Streptococcus* and *Haemophilus* [88,89]. In our study, we observed these two microorganisms in moderate and high dysbiosis groups, respectively, leading us to infer a possible correlation between proteolytic activity and *Streptococcus* and *Haemophilus*. Moreover, ileal *Haemophilus* was positively correlated with faecal *Streptococcus*, indicating a strong link between these two bacteria. Finally, both faecal and ileal *Haemophilus* were positively correlated with intestinal MDI; thus, we can infer *Haemophilus* as a biomarker of intestinal dysbiosis in IBD. It is interesting to note that zeatin biosynthesis is associated with mild MDI. Probably, zeatin could be involved in inflammatory pathways triggered by bacterial pathogens [90]. 

Our results also confirm that there is a distinct and unique GM signature in IBD patients, with a prevalence of pro-inflammatory bacteria associated with high MDI, such as Enterobacteriaceae and *Fusobacterium*, but also protective and immunomodulatory bacteria associated with mild MDI, like *Faecalibacterium*.

In this paper, we presented a novel method for the MDI calculation based on a patent that has not been investigated in any previous published study. The innovation of this method lies in an algorithm based on the comparison of the patients’ gut microbiota profile with that of a group of healthy reference subjects, matched for age with the patients. In fact, there are no studies on gut microbiota that define a reference bacterial profile in healthy paediatric individuals that is able to define a state of intestinal eubiosis. Here, our algorithm is able to define a grade of dysbiosis (MDI) (i.e., mild, moderate and severe).

Furthermore, the correlation of MDI with clinical scores and with disease activity demonstrated the possible application of this analytical parameter in predicting IBD activity and progression.

However, a larger cohort and prospective studies are needed to validate and investigate the potential of the MDI to support IBD clinical management. 

Finally, we have for the first time provided a description of interactions occurring between the mucosal-associated microbiota and the faecal microbiota to advance understanding of the mutual cross-talk between these two ecological niches in this disease.

There are several limitations in this study. Firstly, we could not assess the influence of ileocolonoscopy preparation on the mucosal-associated microbiota composition. However, the use of standardised preparation protocol followed by all recruited patients, would lead one to presume that the evaluation of the mucosal-associated microbiota remains acceptable. Moreover, we mapped the mucosal-associated microbiota by using a single ileal region, and the sample size was comparatively small in this study. Therefore, a large-scale prospective study with multiple intestinal regions is required to confirm our findings. Finally, we could not assess the effects of different alimentary regimens on the composition of gut microbiota, as in our study all patients followed a Mediterranean diet. 

## 4. Materials and Methods

### 4.1. Patient Enrolment 

Paediatric patients with a diagnosis of IBD according to the Porto Criteria [91] were recruited at the Paediatric Gastroenterology and Liver Unit, Sapienza University of Rome [28]. To be included in the IBD group, patients must have met the following criteria: (i) age  ≤  18 years; (ii) had not received antibiotics during the last 2 months; and (iii) had not taken probiotics during the last 2 months. Clinical activity of disease was defined by a Paediatric Crohn’s Disease Activity Index (PCDAI) > 10 and a Paediatric Ulcerative Colitis Activity Index (PUCAI) > 10 for CD and UC, respectively. All the patients followed a dietary regimen comparable to the Mediterranean diet. The patients were recommended to limit their intake of oligo-fructose, lactulose, inulin-containing fruit juices and fibres to avoid alterations in their microbiota composition for 2 weeks prior to biopsy and collection of faecal and urine samples. A questionnaire for the GI symptoms and quality of life was administered the day before the colonoscopy to all the children or their parents [92]. This study was performed in accordance with the principles of the declaration of Helsinki and was approved by the Medical Ethics Committee of Sapienza University (CE: 4032, protocol no.: 281/16). All the parents or legal guardians of the patients gave their signed, informed consent before the enrolment. 

The GM profiles of healthy subjects, present in Bambino Gesù Children’s Hospital digital database, were used to perform GM comparisons between IBD and CTRL and to calculate MDIs.

### 4.2. Sample Collection 

Each patient collected a single faecal sample (35 samples) and urine when possible (24 samples) prior to ileocolonoscopy preparation and stored the samples in their freezer at home within one hour after collection and brought the samples to the hospital in cooled condition. During the ileocolonoscopy, 35 mucosal specimens of non-inflamed terminal ileum tissue were collected and immediately stored at −80 °C. Five mucosal samples were considered unsuitable for sequencing. The unwashed biopsies were sent at a controlled temperature to the Human Microbiome Laboratory of the Children’s Hospital and Research Institute (OPBG) of Rome (Italy) and immediately stored at −80 °C. 

### 4.3. Library Preparation and 16S rRNA Sequencing

Bacterial DNA was extracted from all mucosal and faecal samples as described in [93]. DNA was isolated manually using the QIAmp Fast DNA Stool mini kit (Qiagen, Hilden, Germany) for the faecal samples and automatically using the EZ1 DNA Tissue Kit coupled to the Qiagen EZ1 Advanced XL machine (Qiagen, Hilden, Germany) for ileal biopsies, following manufacturer’s instructions. 

The 16S RNA-targeted metagenomics was performed for all samples. The 16S rRNA V3-V4 hypervariable region (~460 bp) was amplified by using the primers described in the MiSeq rRNA Amplicon Sequencing protocol (Illumina, San Diego, CA, USA). The PCR reaction was set up using the 2× KAPA Hifi HotStart ready Mix kit (KAPA Biosystems Inc., Wilmington, MA, USA) following the manufacturer’s protocol. DNA amplicons were then cleaned up and indexed by a unique combination of Illumina Nextera adaptor-primers. The final libraries were cleaned up, quantified, pooled to a unique library sample and normalised to 4 nM. The following steps consisted of library denaturation and dilution to a concentration of 6.8 pM. To generate paired-end 250 × 2 bp length reads, the library was sequenced on the Illumina MiSeqTM platform according to the manufacturer’s specifications.

### 4.4. Bioinformatic Analysis of 16S Amplicon Sequencing

Analyses were performed with Quantitative Insights Into Microbial Ecology (QIIME2, version 2023.2) [94]. A total of 13,201,597 sequence reads and 2832 Amplicon Sequence Variants (ASVs) from the faecal samples and 3,883,114 sequence reads and 1618 ASVs from the mucosal samples were obtained. A quality filter based on a Phred score > 25 and a denoising and sequence alignment of 99% identity using the DADA2 plugin of QIIME2 were applied [95]. 

The sequences were further aligned to construct a phylogenetic tree with mafft-fasttree via q2-phylogeny [96]. In order to compare the community composition of each sample at a specific taxonomic level, each ASV was taxonomically classified using Greengenes reference database (v13.8, https://greengenes.secondgenome.com/) by means of classify-sklearn naïve classifier via q2-feature-classifier [97]. 

### 4.5. Intestinal MDI Calculation

Using a novel metagenomic method for in vitro diagnosis of gut dysbiosis developed by our algorithm (patent N WO2017216820A1), we were able to assign a degree of dysbiosis in IBD patients compared to gut microbiota profiles from healthy subjects matched for age and gender. According to the patent, metagenomics was used to qualitatively and quantitatively characterise the GM profiles at the phylum, family and genus taxonomic levels. The GM profiles of the patients were then compared with those of healthy subjects stratified by age and gender. Based on the percentage quadratic dissimilarity index Z = (½ × Σ(f_case_ − f_controls_)^2^)^1/2^ × 100 [98] wherein f_case_ was the median value of the taxa distribution at the phylum, family and genus taxonomic levels of GM of a patient and f_controls_ was the median value of taxa distribution at the phylum, family and genus taxonomic levels of GM of healthy subjects. This index varied between 0 and 1 and can be expressed in percentage. A value of 0 indicates no dissimilarity and a value of 1 indicates maximum dissimilarity. This index can therefore be used as a measure of dysbiosis. The degree of dysbiosis was classified as mild (<25%), moderate (25–35%) and high (>35%), according to an empirical algorithm developed during outpatient visit evaluation for treatment of gastrointestinal symptoms.

### 4.6. Statistical Analyses

The count matrix, the taxonomy table and the phylogenetic tree were imported in R v4.1.4 to perform statistical analyses. Ecological analyses of α-diversity and β-diversity were performed on bacterial absolute abundances normalised with the rarefaction method. To compare the α-diversity indexes among several cohorts, the Mann–Whitney test was used, and to verify the statistical significance of inter-dissimilarity group calculations using Bray–Curtis dissimilarity, a permutational analysis of variance (PERMANOVA) test was performed. 

An analysis of confounding factors (age, gender and different treatments) was performed using the confounders function of microbiomeMarker v3.18 [99]. Univariate and multivariate analyses, including linear discriminant analysis effect size (LEfSe) [100] and principal component analysis (PCA), were performed on the ASV relative abundance matrix normalised by the cumulative sum scaling (CSS) method [101] and filtered for bacterial sequences present in less than 25% of the total samples with a relative abundance < 0.01.

Correlation networks between faecal and mucosal communities were built using Spearman’s correlation by means of graph and corrr R packages (v3.18 and v0.4.4, respectively).

Linear regression models were used and Pearson’s correlation analysis was performed to evaluate associations between clinical continuous variables, while logistic regression models were used and a non-parametric test (Kruskal–Wallis and Mann–Whitney test) was applied for categorical variables.

The correlation analysis between ASVs and the intestinal MDI, based on Pearson’s correlation coefficient with the corresponding *p*-value and q-value (*p*-value corrected by the Benjamini–Hochberg FDR procedure [102]) was performed using the corr.test function of the R “psych” package. A positive value of the correlation coefficient (rho-value) indicates a direct correlation between two variables, whereas a negative rho-value indicates an inverse correlation. Rho-values between 0 and 0.3 indicate a weak correlation, those between 0.3 and 0.7 indicate a moderate correlation, and a value greater than 0.7 is defined as a strong correlation [103].

### 4.7. Functional and Network Analyses

Functional pathways were predicted by Phylogenetic Investigation of Communities by Reconstruction of Unobserved States (PICRUSt2) [104] software (https://github.com/picrust/picrust2, accessed on 5 August 2024), using the Kyoto Encyclopedia of Genes and Genomes (KEGG) orthologs database. LEfSe was used to identify statistically significant biochemical pathways (α-value of 0.05 and a logarithmic linear discriminant analysis (LDA) score threshold of 3.0). 

### 4.8. Intestinal Permeability, Mucosal Immunity Activation and Metabolic Dysbiosis Analyses

Faecal zonulin levels (Zpn) and faecal secretory IgA (sIgA) levels were measured by enzyme-linked immunosorbent assay (ELISA) kits (Immundiagnostik AG, Bensheim, Germany), according to the product instructions. The absorbance for both tests was measured at 450 nm, using a microplate reader. 

Indican (indoxyl sulphate) was quantitatively measured in urine samples by a QuantyChrom TM Indican Assay Kit (Biossay Systems, Hayward, CA, USA), following the manufacturer’s instructions. The absorbance was measured in a microplate reader at 480 nm. 

## 5. Conclusions

We are able to consider the following as biomarkers of GM: Enterobacteriaceae, *Fusobacterium*, *Haemophilus* and *Veillonella* in IBD; and faecal Gemellaceae and Enterobacteriaceae and ileal *Fusobacterium*, *Haemophilus* and *Veillonella* in IBD patients with a high MDI.

Moreover, faecal Enterobacteriaceae and ileal *Haemophilus*, *Actinobacillus* and *Prevotella* were positively correlated with MDI. 

In addition, we found biomarkers of active disease: Ruminococcaceae and WAL_1855D; the latter was also positively correlated with MDI. Furthermore, the MDI result correlated with PUCAI and Matts scores.

In conclusion, the MDI showed a strong association with both severity and activity of IBD and a positive correlation with clinical scores, especially in UC. Moreover, markers of metabolic dysbiosis, intestinal permeability and mucosal immunity activation deserve to be included in further studies to deepen the interaction amongst GM, immunity and inflammatory processes in IBD.

## Figures and Tables

**Table 1:** Demographical and clinical characteristics of the 35 IBD patients.

Clinical Features	N (%)	
**Gender, male/female (%)**	18/17 (51.4/48.6)	
**Age, mean (years)**	14.4	
	Treatment	
**5-ASA ^1^**	16 (45.71)	
**Antibiotics**	3 (8.57)	
**Immunosuppressants**	10 (28.57)	
**Biological therapies**	11 (31.42)	
	Disease activity	
**Active**	11 (31.4)	
**Remission**	24 (68.6)	
	Disease severity	
**Remission**	16 (45.7)	
**Mild**	14 (40)	
**Moderate**	5 (14.3)	
	Disease localisation	
**Absence**	4 (11.43)	
**Proctitis**	3 (8.57)	
**Left colitis**	4 (11.43)	
**Extensive colitis**	9 (25.71)	
**Ileo/Ileocolon**	15 (42.8)	
	IBD conditions	
	UC (n = 14)	CD (n = 21)
**Activity index**	PUCAI N (%)	PCDAI N (%)
**Remission (<10)**	4 (28.6)	12 (57.1)
**Mild (10–34)**	10 (71.4)	6 (28.6)
**Moderate (35–64)**	NA	3 (14.3)
**Severe (>65)**	--	--

^1^ 5-amminosalicilic acid. NA: Not Available.

**Table 2:** Pearson’s correlation test between bacteria and intestinal MDI.

Faecal Microbiota	Linear Correlation Coefficient	*p*-Value	q-Value
Enterobacteriaceae	0.634	0.00004	0.00217
*Fusobacterium*	0.435	0.00908	0.44492
*Haemophilus*	0.399	0.01745	0.80292
WAL_1855D	0.337	0.04738	1.00000
Lachnospiraceae_*Clostridium*	−0.422	0.01152	0.55273
*Bacteroides*	−0.409	0.01474	0.69299
*Butyricicoccus*	−0.392	0.02000	0.89989
*Faecalibacterium*	−0.537	0.00087	0.04341
**Ileal Microbiota**			
*Achromobacter*	0.4017	0.02778	1.00000
*Actinobacillus*	0.4547	0.01159	0.59126
*Cloacibacterium*	0.4832	0.00684	0.35559
*Haemophilus*	0.4507	0.01244	0.62188
*Prevotella*	0.4257	0.01901	0.93166
*Pseudomonadaceae*	0.4230	0.01985	0.95267

## Data Availability

The dataset presented in this study can be found in online repositories. The name of the repository/repositories and accession number(s) can be found below: PRJNA1136812 (https://www.ncbi.nlm.nih.gov/bioproject). Further inquiries can be directed to the corresponding authors.

## Supplementary Materials

The following supporting information can be downloaded at https://www.mdpi.com/article/10.3390/ijms25179618/s1.
