# Supplementary material for: A Novel Microbial Dysbiosis Index and Intestinal Microbiota-Associated Markers as Tools of Precision Medicine in Inflammatory Bowel Disease Paediatric Patients
Source: Int J Mol Sci. 2024 Sep 5;25(17):9618. doi: 10.3390/ijms25179618 (PMC11395508; doi:10.3390/ijms25179618)
Supplement: Supplementary file 1 [file ijms-25-09618-s001.zip › ijms-3170139-supplementary.pdf]

Supplementary Material

Figures

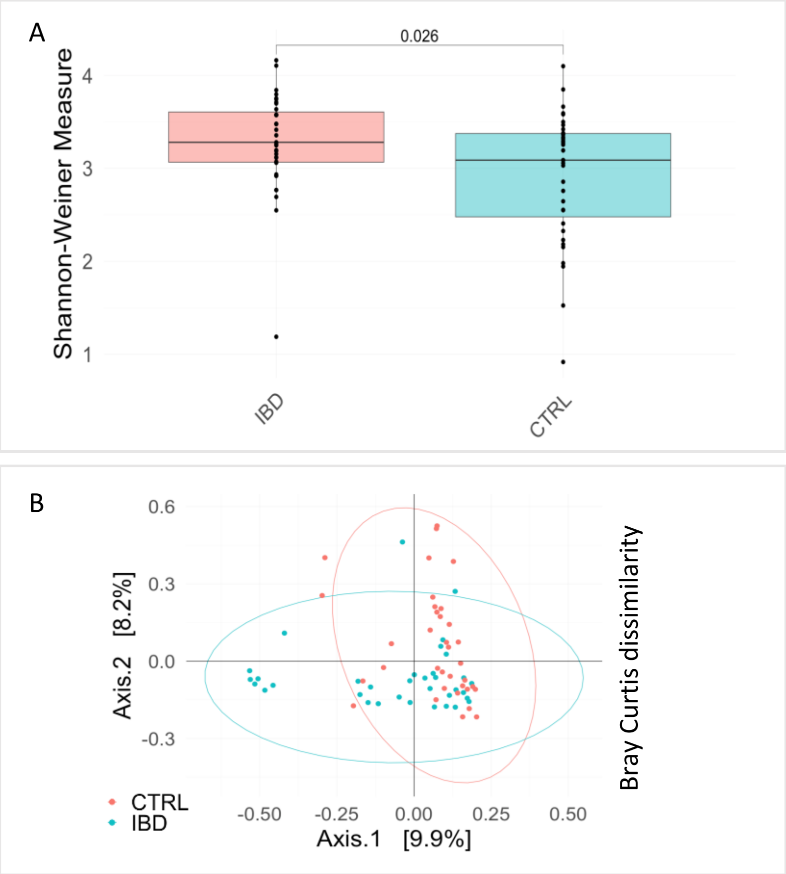

Figure S1. Ecological analysis of gut microbiota from the comparison between IBD patients and CTRLs. (**A**) Alpha diversity, based on the Shannon-Weiner index. The Mann-Whitney U test was resulted statistically significant, *p*-value = 0.026. (**B**) Beta-diversity was calculated using the Bray-Curtis algorithm. PERMANOVA test was not statistically significant, *p*-value > 0.05.

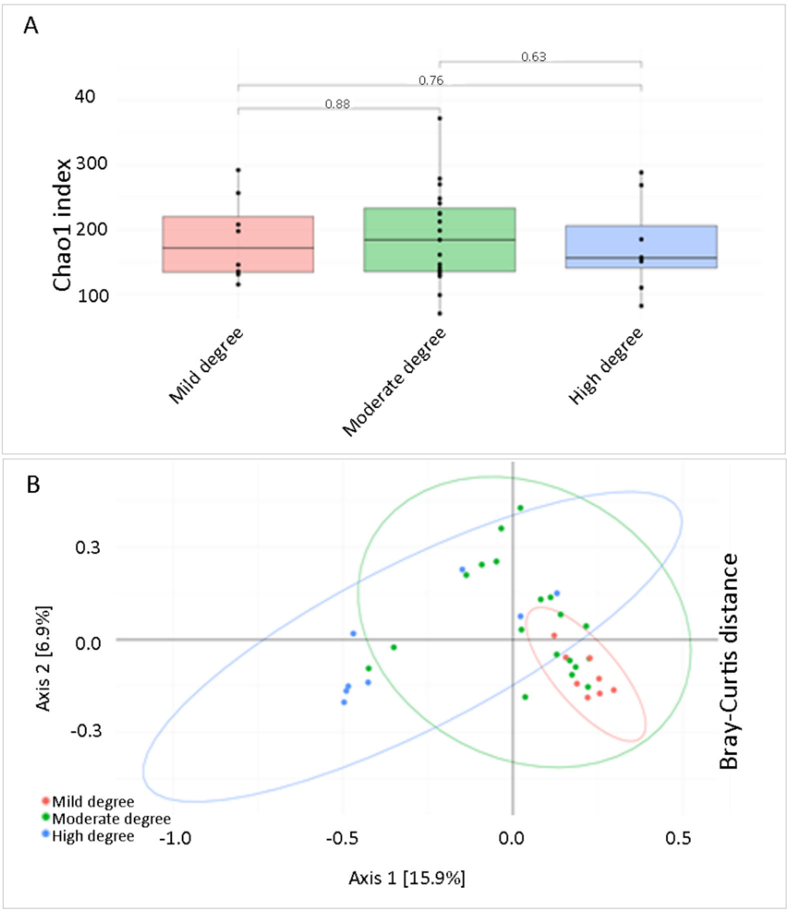

Figure S2. Ecological analysis of gut microbiota from IBD patients stratified for Microbial Dysbiosis Index (MDI) degree. (**A**) Alpha diversity, based on the Chao1 index. The Mann-Whitney test, applied for the comparisons between IBD patients with mild, moderate and high dysbiosis, was resulted not statistically significant. (**B**) Beta-diversity was calculated using the Bray-Curtis algorithm. PERMANOVA test was statistically significant, *p*-value = 0.008.

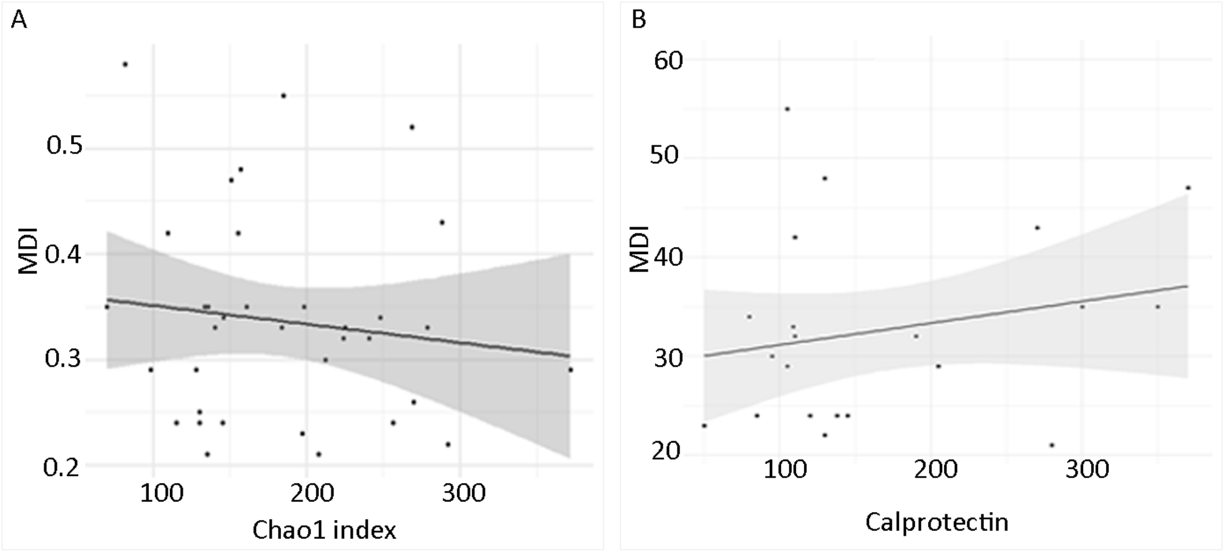

Figure S3. Fitted line plot of intestinal MDI and Chao1 index (A) and faecal calprotectin levels (B) in IBD patients. Regression analysis showed no correlation between these two variables (p-value=0.47 and p-value =0.29, respectively).

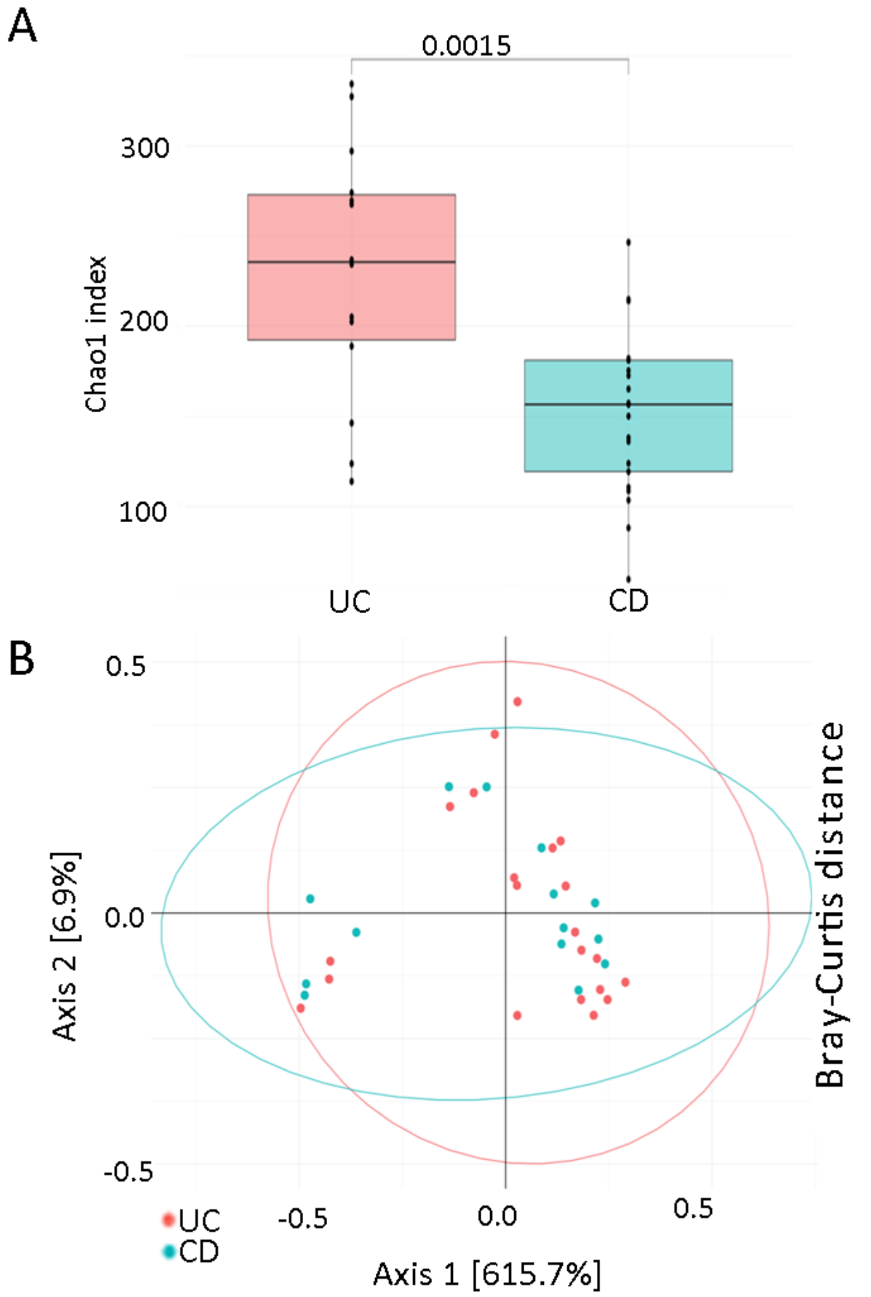

Figure S4. Ecological analysis of gut microbiota from IBD patients stratified for CD and UC. (**A**) Alpha diversity, based on the Chao1 index. The Mann-Whitney test, applied for the comparisons between CD and UC, was resulted statistically significant (p=0.0015). (**B**) Beta-diversity was calculated using the Bray-Curtis distance algorithm. PERMANOVA test was not statistically significant, PERMANOVA>0.05.

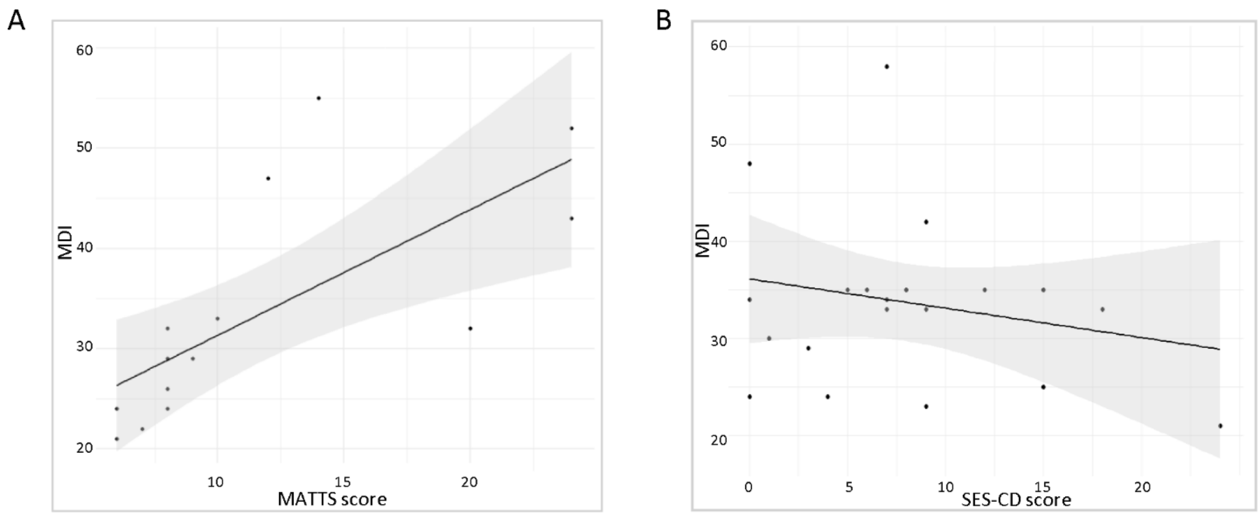

Figure S5. Fitted line plot of intestinal MDI and MATTS score index in UC (A) and SES-CD score in CD (B). Intestinal MDI resulted correlated with Matts score in UC patients, p=0.004 **(A).** The correlation between gut MDI and SES-CD in CD patients resulted not statistically significant, p=0.35 (**B**).

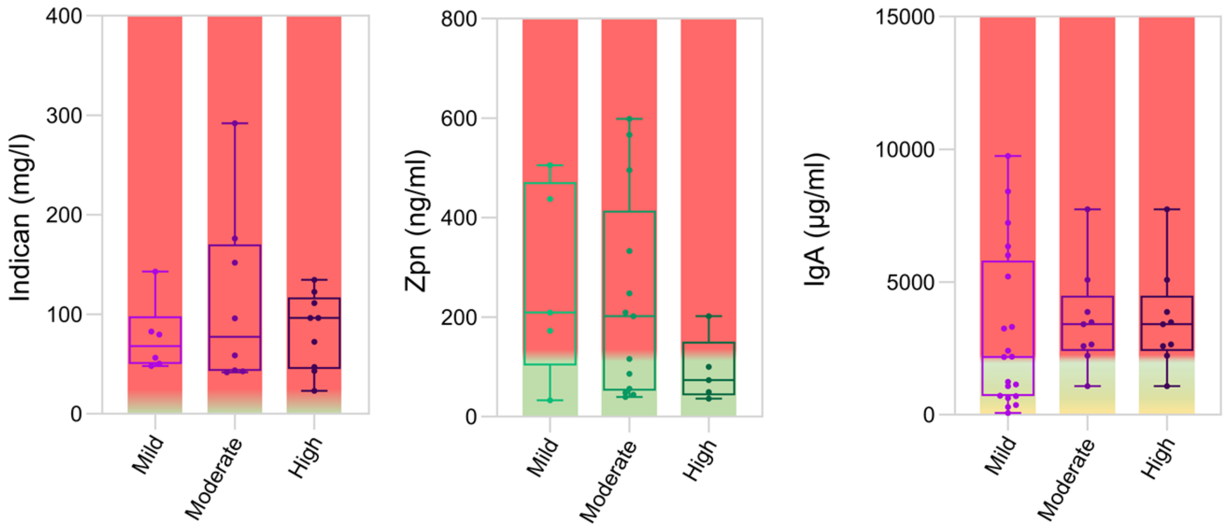

Figure S6. Metabolic dysbiosis, intestinal permeability and mucosal immunity activation in relation to MDI. Boxplots of indican, Zpn and IgA levels in IBD patients stratified for MDI subgroups. The one-way ANOVA test was used for subgroup comparisons. For all comparisons p-values>0.05.

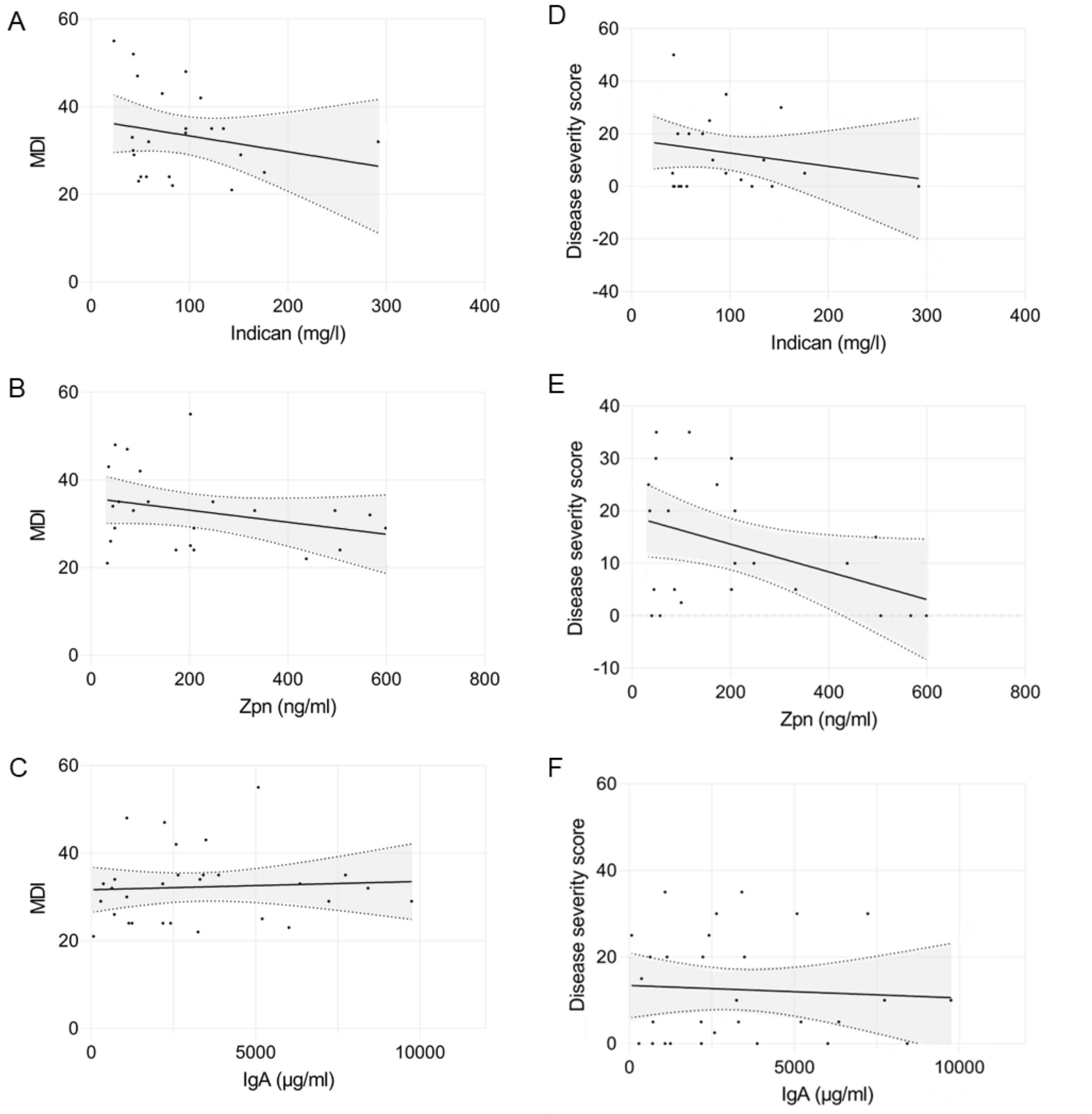

Figure S7. Linear regression between MDI and dysbiosis (A), permeability (B) and mucosal immunity activation (C) parameters in IBD patients. (A) There is no statistically significant correlation between MDI and urinary indican levels, (p-value=0.31). **(B)** No statistically significant correlation between MDI and zonulin (Zpn), (p-value=0.18). **(C)** No statistically significant correlation between MDI and faecal IgA levels, (p-value=0.75). **Linear regression between disease severity score and dysbiosis (D), permeability (E) and mucosal immunity activation (F) parameters in IBD patients. (D)** There is no statistically significant correlation between disease severity score and urinary indican levels, (p-value=0.34). **(E)** No statistically significant correlation between disease severity score and zonulin (Zpn), (p-value=0.05). **(F)** No statistically significant correlation between disease severity score and faecal IgA levels, (p-value=0.74).

Tables

Table S1. Results of Mann-Whitney U-test for comparison between IBD and CTRL.

	IBD	CTRL	P value	FDR p value
Coprococcus	0.008	0.014	0.002	0.000
Oscillospira	0.019	0.031	0.000	0.000
Clostridiales	0.011	0.066	0.000	0.000
Ruminococcaceae	0.022	0.063	0.000	0.000
Akkermansia	0.045	0.168	0.000	0.000
Haemophilus	0.026	0.000	0.000	0.000
Christensenellaceae	0.001	0.006	0.000	0.000
Ruminococcaceae Ruminococcus	0.028	0.055	0.003	0.000
Alistipes	0.009	0.021	0.002	0.000
Gemmiger	0.026	0.049	0.000	0.000
Gemellaceae	0.003	0.000	0.000	0.000
Streptococcus	0.026	0.008	0.005	0.000
Mogibacteriaceae	0.002	0.005	0.000	0.000
Eggerthella	0.004	0.001	0.000	0.000
Enterobacteriaceae	0.127	0.044	0.001	0.000
Methanobrevibacter	0.000	0.002	0.001	0.000
Ruminococcus	0.046	0.011	0.002	0.000
Rikenellaceae	0.001	0.010	0.000	0.000
Enterococcus	0.025	0.002	0.001	0.000
Anaerostipes	0.003	0.002	0.006	0.022
Barnesiellaceae	0.009	0.014	0.008	0.022
Parabacteroides	0.005	0.017	0.006	0.022
Lactobacillus	0.006	0.000	0.005	0.022
Sutterella	0.012	0.001	0.010	0.022
Prevotella	0.002	0.022	0.023	0.040
Fusobacterium	0.003	0.000	0.016	0.040

Table S2. Confounding factors analysis performed by microbiomeMarker.

Confounder	pseudo_F	p-value
**Biologic**	1.078	0.174
**Immunosuppressant**	1.131	0.089
**5-ASA**[1]	1.007	0.445
**Antibiotic**	1.018	0.426
**Age**	1.127	0.148
**Gender**	1.018	0.350

[1] 5-amminosalicilic acid

Table S3. Comparison of MDI in patients stratified for disease localization (Mann-Whitney test).

Localization Group 1	Localization Group 2	p-value
**Proctitis**	Left colitis	1
**Proctitis**	Extensive colitis	0.578
**Proctitis**	Ileo/Ileo-Colon	0.472
**Left colitis**	Extensive colitis	0.215
**Left colitis**	Ileo/Ileo-colon	0.06
**Extensive colitis**	Ileo/Ileo-colon	1
